# Rapid neonatal AAV delivery for adult cortical two-photon imaging of genetically encoded sensors

**DOI:** 10.1016/j.isci.2025.113898

**Published:** 2025-10-30

**Authors:** Stephen Wisser, Ethan B. Blackwood, Kallol Bera, Andrzej Z. Wasilczuk, Loren L. Looger, Alex Proekt, Joseph Cichon

**Affiliations:** 1 Department of Anesthesiology and Critical Care, Perelman School of Medicine, University of Pennsylvania, Philadelphia, PA, USA; 2 Department of Neuroscience, Perelman School of Medicine, University of Pennsylvania, Philadelphia, PA, USA; 3 Howard Hughes Medical Institute, University of California, San Diego, La Jolla, CA, USA; 4 Department of Neurosciences, University of California, San Diego, La Jolla, CA, USA

**Keywords:** Behavioral neuroscience

## Abstract

Elucidating the mechanisms of the central nervous system necessitates the ability to precisely visualize, record, and manipulate specific neuronal populations within the living brain. Here, we report a fast and efficient technique for delivering adeno-associated virus (AAV) in neonatal pups for the robust co-expression of reporters and sensors in cortical neuronal subpopulations under different promoters and transgenic mouse lines. This approach has lower training requirements than adult AAV injections, and it enables a 40- to 50-fold increase in injection throughput per animal with high reproducibility. Pup-injected adult mice show reduced signals of neuroinflammation compared to adult-injected mice. Importantly, adult pup-injected mice have stable and high expression of multiple AAVs suitable for structure-function two-photon imaging in local cortical circuits of awake, behaving adult animals. This approach offers a fast, efficient, and robust method of introducing multiple AAVs for investigating neuronal structure and function in defined cortical neuronal populations in the living brain.

## Highlights

• Neonatal AAV injections are quicker and require less training than adult injections
• Pup injected adults show reduced neuroinflammation
• Cells stably co-express up to 4 AAVs
• Enables *in vivo* microscopy of both individual neurons and large neuronal populations

## Graphical abstract

**Figure:**
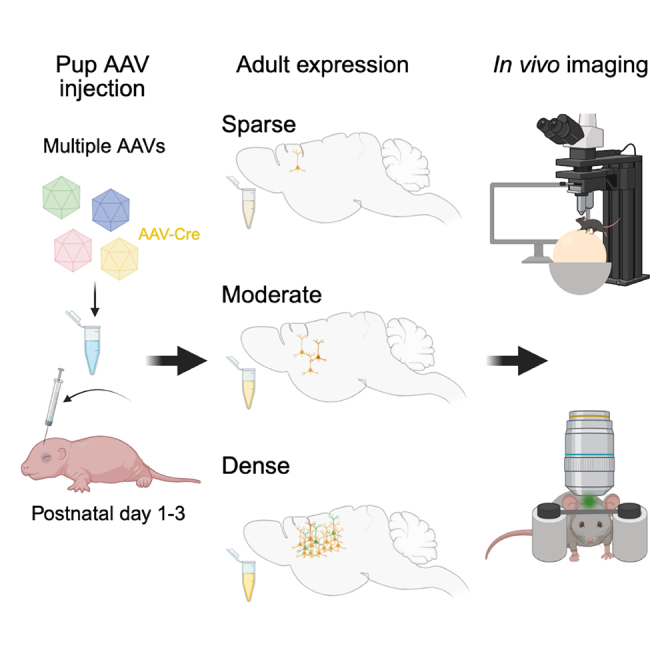

## Introduction

*In vivo* imaging of neuronal activity, coupled with cell-type-specific manipulations, is a powerful technique for advancing our understanding of microcircuits in the living brain. A key factor in the success of these approaches is the robust and specific expression of multiple genetically encoded activity reporters and actuators, often within individual cells.^1^^,^^2^^,^^3^^,^^4^ This expression is typically accomplished through either transgenic animals or adeno-associated virus (AAV)-mediated transduction of the genes of interest. Transgenic animals provide widespread stable sensor expression for the lifetime of the organism,^5^^,^^6^ eliminating the need for invasive survival surgeries for sensor delivery. However, transgenic models often exhibit weaker sensor expression compared to adeno-associated virus (AAV) transduction strategies^7^ and are typically more costly and time-intensive, making them prohibitive for many researchers.^8^^,^^9^^,^^10^^,^^11^ Consequently, the use of adult AAV injections has become an increasingly common approach for sensor expression in the living brain.^12^^,^^13^

AAV-mediated expression of genetically encoded indicators for neuronal activity and actuators is a rapid and cost-effective method, enabling region-specific expression^8^^,^^14^ with long-term transgene stability that can persist for years in some animals.^15^^,^^16^^,^^17^ Currently, AAVs are routinely delivered via stereotaxic intraparenchymal injection in adult rodents.^18^^,^^19^ While effective, this approach requires survival surgery and the insertion of an electrode or syringe for AAV delivery. As a result, adult AAV expression involves significant technical training and equipment for anesthesia and surgery, potential damage as the injection itself may induce inflammation and direct trauma, and time for post-injection expression.^20^^,^^21^^,^^22^

To circumvent some of the challenges associated with adult stereotaxic surgeries while keeping the benefits of AAV expression, alternative AAV delivery routes have been introduced. Intravenous (IV) delivery—particularly via the transverse sinuses^23^^,^^24^^,^^25^ and retro-orbital sinuses^21^^,^^26^^,^^27^—as well as intracerebroventricular (ICV) injections of viral vectors have proven effective in driving widespread expression throughout the CNS.^13^^,^^21^^,^^23^^,^^24^^,^^25^^,^^26^^,^^27^^,^^28^ These approaches are fast, non-invasive, do not require surgical expertise, and provide the expression of fluorescent reporters for at least 1 year.^13^^,^^19^^,^^29^ However, AAV delivery through both blood and CSF typically requires large volumes of virus to induce appropriate expression,^30^ and IV administration of AAV is associated with a heightened immune response.^31^ Furthermore, AAVs delivered through CSF do not ensure expression is confined to the CNS,^32^ and when it is, expression is often not uniform within superficial regions, as regions adjacent to CSF spaces receive a greater expression bias.^30^^,^^32^^,^^33^ This feature is of particular concern for *in vivo* two-photon imaging experiments where superficial cortical imaging (layers 2/3 to 5) may be desired. While this regional tropism effect of CSF delivery methods may be adequately controlled by using the appropriate serotype,^13^^,^^29^ a direct AAV injection may be more efficient.

To this end, we have developed a method for direct intraparenchymal injection of AAV vectors into neonatal mouse brains for the stable expression of a variety of genetically encoded reporters and sensors. Compared to typical adult intraparenchymal injection surgeries, our neonatal approach significantly reduces the time needed to accomplish an individual brain injection of multiple AAVs by 40–50 times, reduces anesthesia and recovery times, and is accompanied by a mitigated neuroinflammatory response. The resulting adult animal reliably coexpresses up to 4 AAVs for *in vivo* two-photon laser scanning microscopy. Given the speed at which our technique can be mastered, our neonatal intraparenchymal injection method may serve as an efficient way to capture the advantages of the AAV-mediated expression of genetically encoded sensors while eliminating most of the challenges and limitations associated with adult AAV injections for cortical-based imaging studies.

## Results

### Adeno-associated virus injection into neonatal pups requires less time and training than adult injections

**Figure 1:**
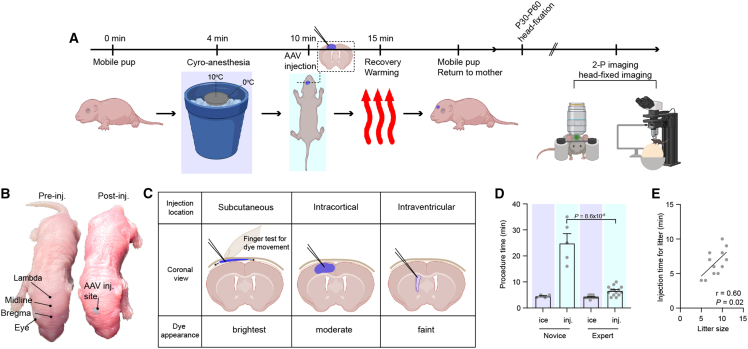
Neonatal intracranial injection is a fast and efficient method for AAV introduction (A) Experimental timeline for neonatal AAV intracranial injection. Pups can achieve immobility within 4 min after exposure to ice treatment. A litter of pups (avg. litter size: 7.8 ± 0.5) can be injected within ∼6 min. Pups are subsequently slowly warmed on a heating pad. Once a pup regains mobility, they are placed back with their mother for further recovery and development. (B) Critical visual landmarks (left) to aid AAV delivery. The AAV/fast green dye mix was specifically targeted at the medial prefrontal cortex. (C) Table describes the injectate location and appearance of the dye. AAV/f-dye located between the scalp and skull; the dye is brightest, and the injectate can move freely when the experimenter’s finger can move the dye across the midline/hemispheres. If the injectate is deep into the cortex, the dye will appear faint and might fill the lateral ventricle. Moderate intensity of dye would signal an intracortical injection. (D) Procedure time for injecting an entire litter based on skill level. With increasing practice, an experimenter can obtain increased efficiency (novice vs. expert: *p* = 8.6 × 10-5, Mann-Whitney test). Expert-level skills will also enable single-injection success. Data are represented as mean +SEM. (E) Procedure time is positively correlated with litter size only by a few minutes (Pearson correlation: r = 0.60, *p* = 0.02). See also Figure S1.

To stably express genetically encoded activity reporters in distinct neuronal cell types in cortical circuits, we have developed a simplified approach to virally deliver transgenes in a region-specific manner to the neocortex of neonatal mice (Figure 1A). Neonatal pups are best suited to AAV injection during postnatal (P) days 0–3, with P0-1 being superior to P3 because landmarks can be identified through transparent skin (Figure 1B). While injections can be performed at P3, skin changes, including darkening and thickening, that come with normal mouse development make immediate visualization of injection success less clear (Figure S1). We observed suitable landmarks in both C57BL/6J and CD-1 mice up to P3. Rigorous daily monitoring of pregnant breeders assists in the accurate birth dates of litters. First-time mothers or older mothers tend to neglect pups, which typically leads to higher mortality post-injection. Neonatal pups well suited for injection have milk visible in their stomach, which aids in perioperative recovery and serves as a nutritional bridge until nursing is re-established. In preparation for injection, glass micropipettes (Drummond, 5-000-1001-X10) should be pulled and beveled to a ∼30–45° angle. A plunger should be lightly coated with mineral oil to enable the AAV solution to be drawn up and injected with microliter precision. We routinely mix AAV stock (∼100–500 nL of high titer virus, typical titer ≥7×10^12^ vg/mL) with a small volume of highly concentrated biocompatible dye (Fast Green; f-dye) to report the location and extent of injectate in real-time (Figures 1B and 1C, see later in discussion). The dye spontaneously clears by the following day.

On procedure onset, pups are briefly rendered immobile for injection by hypothermia. An aluminum T-shaped block is placed on a bed of ice for 10 min before the procedure starts, bringing its temperature to ∼10°C at the center and 0°C at the edges. Pups placed in the middle of the block slowly become immobile, reaching full immobility by ∼4 min. Note that pups positioned at the edges are at risk of frostbite. P3 pups may require extended cooling times due to their relatively thicker skin. Once immobile, the pup’s head is cleaned with an alcohol swab and placed under an upright stereomicroscope, enabling experimenters to visualize critical injection landmarks (Figure 1B). Key landmarks to help orient experimenters prior to injection include the developing eyes, bregma, lambda, and the frontal and midline sinuses. Thus, depending upon the region of interest (ROI) for adult two-photon imaging, the AAV/f-dye mix can be directed to unilateral or bilateral cortical regions (Figure S1). After identifying an ROI, the experimenter holds a micropipette filled with AAV/dye in their dominant hand and uses their non-dominant hand to stabilize the pup’s head as the dominant hand gently guides the micropipette to penetrate the skin and skull. Once the micropipette penetrates both the skin and skull (felt as one pop), the experimenter depresses the plunger to deliver ∼100–1000 nL of the virus mix (typical volume, 300–500 nL). While depressing the plunger, it is critical that the micropipette’s position remains stable to avoid depositing the AAV/dye deeper into the brain, potentially into the ventricles. The pipette is then vertically retracted, and the pup is moved to a warming pad. Once mobile, the pup is then returned to the mother. While this protocol describes the freehand approach for AAV targeting and delivery, the pup’s head and body could be held in a stereotactic frame to minimize positioning variability from injection to injection across the litter.^34^ We found that the freehand approach was sufficient for targeting common two-photon imaging regions across the mouse brain, with minimal variability between injections.

The success of the injection can be determined instantly by examining the f-dye appearance under the stereomicroscope (Figure 1B). When the dye appears brightest and spreads widely from the injection site, the AAV/f-dye is likely subcutaneous (i.e., between the scalp and developed skull), an undesired outcome. In this case, the injectate moves freely in all directions and across hemispheres if the injection location is close to midline (Figure 1C). The fixedness of the injectate in the vicinity of the injection site can be assessed by gently rolling the finger over the region (the “finger test”) (Figure 1C). Note that an injection can be re-attempted, although landmarks will not be as apparent after the first injection attempt. If the f-dye appears moderately bright and passes the finger test, this is a successful intracortical injection. If the f-dye appears faint, this may indicate either a subcortical or ventricular injection based on the experimenter’s region of interest, and pups can undergo a re-attempted injection with preserved landmarks. Note that intraventricular injection will allow AAV to be spread in the CSF system to label diverse regions, including the cortex, but will dilute the expected labeling density. If multiple off-site injections occur, experimenters would be encouraged to acutely euthanize the pup to prevent further experimentation on those animals given unpredictable AAV labeling. By euthanizing the pup at this point, the experimenter can reduce unsuccessfully injected mice mixing in with successfully injected mice. We have found that novice experimenters require time to learn the injection location and f-dye appearance, often requiring multiple attempts to achieve successful targeting of the cortex (Figure 1D). Within ∼5 attempts at this procedure, experimenters accelerate their speed to first injection success, with only the litter size becoming a limiting time factor (Figures 1D and 1E).

**Figure 2:**
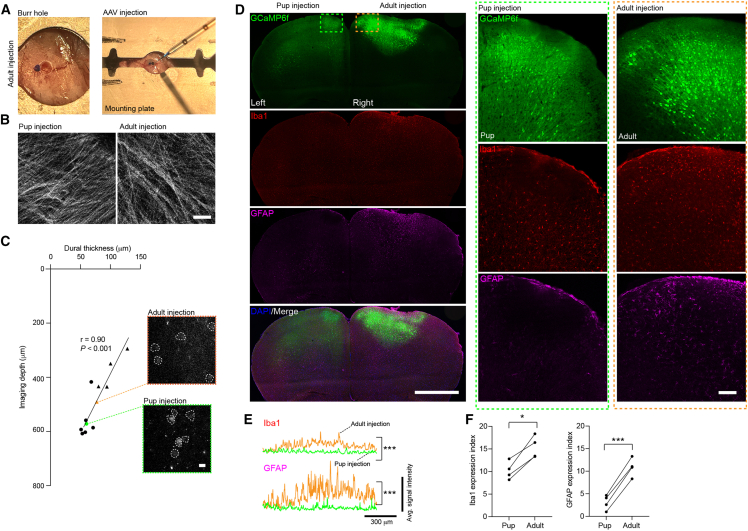
Neonatal AAV injection minimizes glial activation as compared to adult AAV injection (A) Photograph of an anesthetized mouse undergoing adult AAV injection. A metal headplate is affixed to the skull, and a burr hole is drilled at the target location. A glass micropipette is then used to gently penetrate the thinnest portion of the burr hole and is lowered to the desired depth. AAV is delivered slowly over a 10–20 min period to ensure precise and controlled injection. (B) Two-photon z-stacks of the dura in adult mice injected with AAVs either neonatally or in adulthood. Scale bars, 100 μm. (C) Dural thickness is strongly anticorrelated with maximum two-photon imaging depth in mice injected with AAV9-*hSYN*-GCaMP6f as pups (circles) or adults (triangles), with thinner dura associated with deeper imaging (Pearson correlation: r = 0.90, *p* < 0.001). Right, representative two-photon GCaMP6f images from adult (top; orange triangle from left graph) and pup-injected mice (bottom; green circle from left graph) at their respective cortical depths. Scale bars, 20 μm. (D) Left, coronal sections of the prefrontal cortex of an adult mouse that underwent neonatal AAV9-*hSYN-*GCaMP6f injection (left hemisphere) followed by adult AAV9-*hSYN-*GCaMP6f injection (right hemisphere). Sectioned tissue was immunostained against Iba1, GFAP, and DAPI. Scale bars, 1 mm. Right, dashed boxed regions from the left panels highlight increased Iba1 and GFAP immunoreactivity in the adult-injected hemisphere (orange boxed region) compared to the neonatally (green boxed region) injected hemisphere. Scale bars, 100 μm. (E) Average fluorescent intensity measurements of Iba1 and GFAP immunoreactivity across GCaMP6f-expressing regions reveal a significant increase in signal intensity in adult AAV-injected hemisphere mice (orange lines) compared to the pup-injected hemisphere (green lines) (Top: repeated measures aligned rank transform (ART) ANOVA: main effect of injection method, F = 3779.052, *p* < 0.001, Bottom: repeated measures ART ANOVA: main effect of injection method, F = 3925.961, *p <* 0.001). Lines represent the mean intensity from 4 animals. (F) Iba1 (left) and GFAP (right) expression indices from individual mice in (E). Pup-injected hemispheres show significantly less Iba1 (paired *t* test: t = 5.539, *p* = 0.0116) and GFAP (paired *t* test: t = 19.97, *p* = 0.0003) immunoreactivity relative to their contralateral adult-injected hemisphere. See also Figures S2–S4.

The conventional approach to AAV expression in the cortex is direct intraparenchymal injection into adult mice.^18^^,^^19^ This approach requires general anesthesia, surgery (mounting in stereotactic frame, incision, craniotomy/burr hole, suture), a slow period of AAV delivery via nanoinjector or picospitzer (pipette loading and injection), and recovery (anesthesia, pain control) (Figure 2A). Even an experienced experimenter typically requires ∼35-45 min to inject a single mouse.^35^ In contrast, the pup approach takes approximately 47 s, representing a ∼40- to 50-fold reduction in time per animal.

### Neonatal adeno-associated virus injection shows reduced signs of neuroinflammation compared to adult injection

To further evaluate the suitability of this approach for capturing structure-function changes in cortical neurons, we performed two-photon imaging on pup-injected animals over postnatal days 30–60 (Figure 1A). Before two-photon imaging, injected animals showed normal development and comparable performance on various behavioral tests relative to non-injected adult controls, indicating no observable abnormalities (Figure S2). In preparation for two-photon imaging, animals undergo an anesthetic event for head bracket implantation, followed by recovery and head restraint habituation sessions. We have appreciated that when implanting pup-injected mice, their skull surface has no evidence of neonatal injection (Figure S3). Adult-injected mice often still have evidence of craniotomy (bone defect with glue remnants), bony regrowth, and increased connective tissue at neighboring locations around the injection (Figure S3). When implanting the glass cranial windows, we noticed that pup-injected mice have a normal-appearing dura (no sign of proliferation) and intracranial blood vessel architecture. In contrast, adult-injected mice may have dural thickening and blood vessel changes related to electrode/syringe placement (Figures 2B and 2C). On average, two-photon imaging achieved greater depth in pup-injected mice compared to adult-injected mice, likely due in part to reduced dural thickening^36^ (Figure 2C).

Postsurgical changes after adult AAV injection could be sensed by non-neuronal cells, which respond to states of neuroinflammation. To assess this possibility, we examined the activation of astrocytes and microglia, as indicated by the increased expression of glial fibrillary acidic protein (GFAP) and ionized calcium-binding adaptor molecule 1 (Iba1), respectively, in individual mice subjected to both pup and adult AAV9-hSyn-GCaMP6f injections targeting different hemispheres (Figure 2D). AAV-mediated GCaMP6f expression in the adult-injected hemisphere seemed to be more restricted in spread, with a brighter fluorescent signal relative to the pup-injected side. However, we observed a significant increase in GFAP and Iba1 signals in the cortical areas surrounding the adult-injected side as compared to the contralateral pup-injected side (Figures 2D–2F). Moreover, two-photon imaging of calcium signals in astrocytes under the glial-specific promoter *GfaABC1D* revealed that adult-injected mice exhibited significantly more large, spontaneous, multi-ROI calcium transients across individual astrocytes in the prefrontal cortex (PFC) compared to pup-injected mice (Figure S4). Pup-injected mice exhibited glial labeling with extended (flat-appearing) processes, in contrast to the contracted morphology observed in adult-injected mice. Additionally, they allowed for imaging at deeper focal planes, likely due to deeper laser penetration (Figure S4). Since calcium signals in astrocytes may reflect local changes in the consumption of energy, circuit activity, and even brain states, these results raise the possibility that the activation of the glia with adult AAV injection may be linked to changes in cellular and circuit properties that could alter *in vivo* imaging studies of neuronal cells.

### Neonatal injection enables single- to multi-adeno-associated virus expression in individual neurons for *in vivo* two-photon microscopy

**Figure 3:**
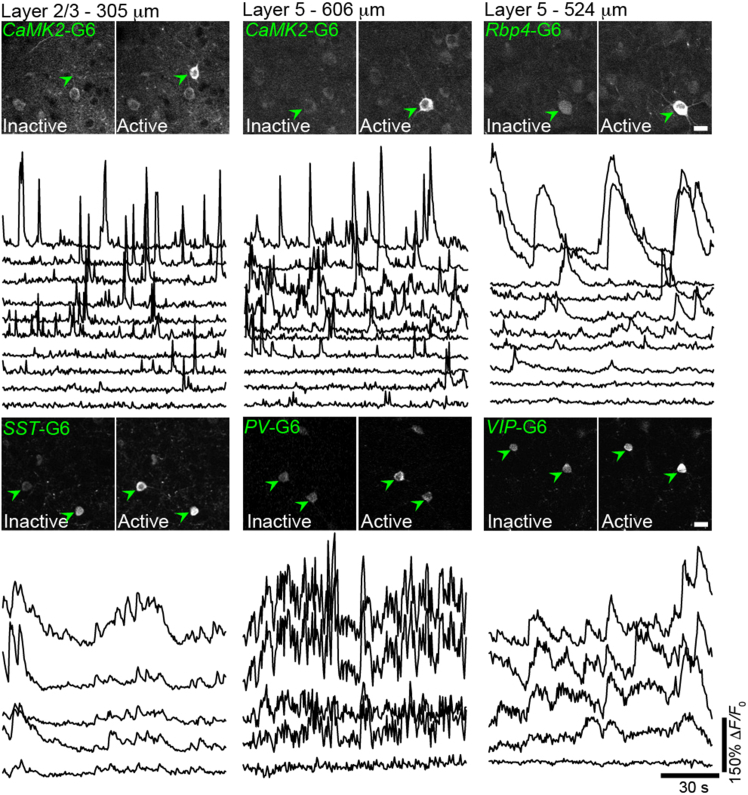
Neonatal AAV injection results in robust adult GCaMP6 expression in various neuronal cell types Two-photon images (top panel) and representative GCaMP6f fluorescent traces (bottom panel) of spontaneous neuronal activity from pyramidal neurons (CaMKII-expressing layer 2/3 and layer 5 neurons and Rbp4-expressing layer 5 neurons) and GABAergic interneurons (SST, PV, and VIP subtypes) in the prefrontal cortex of awake, head-restrained adult mice. Active cells (peak signal images shown in right panels) are denoted by a green arrowhead. Cre-dependent AAVs were injected at P1. Scale bars, 20 μm. See also Figure S5.

To examine the expression of individual AAV constructs injected into transgenic pups, adult animals were prepared for awake animal imaging as previously described.^37^^,^^38^ Two-photon imaging of GCaMP6f in different transgenic lines revealed the robust expression of the sensor in diverse cell types (Figure 3). Recordings in either glutamatergic neurons or GABAergic interneurons over minutes revealed strong spontaneous calcium responses in the PFC. GCaMP expression appeared to be cytoplasmic and excluded from the nucleus, indicating normal intracellular expression. Pup transduction enabled deep labeling, as cell bodies in pyramidal neurons and interneurons could be visualized and recorded at depths of >500 microns from the pial surface (Figure 3). We also observed that the expression of a structural reporter was strong and adequate for subcellular and cellular imaging superficially and deep into the cortex (Figure S5).

We next examined the co-expression of two AAV constructs using AAV9-*CaMKII*-Cre and AAV9-Flex-GCaMP6f delivered to neonatal pups at a ratio of 1:5. Awake, behaving adult mice imaged at P30-50 exhibited robust labeling at superficial L2/3 to deep L5 imaging depths (Figures 3 and S5). We also assessed the delivery of AAV-Cre with other Cre-dependent AAVs, cFos-GFP and DREADD-hM3D(Gq)-mCherry, and found similar expression and labeling across cortical layers in the PFC (Figure S5).

**Figure 4:**
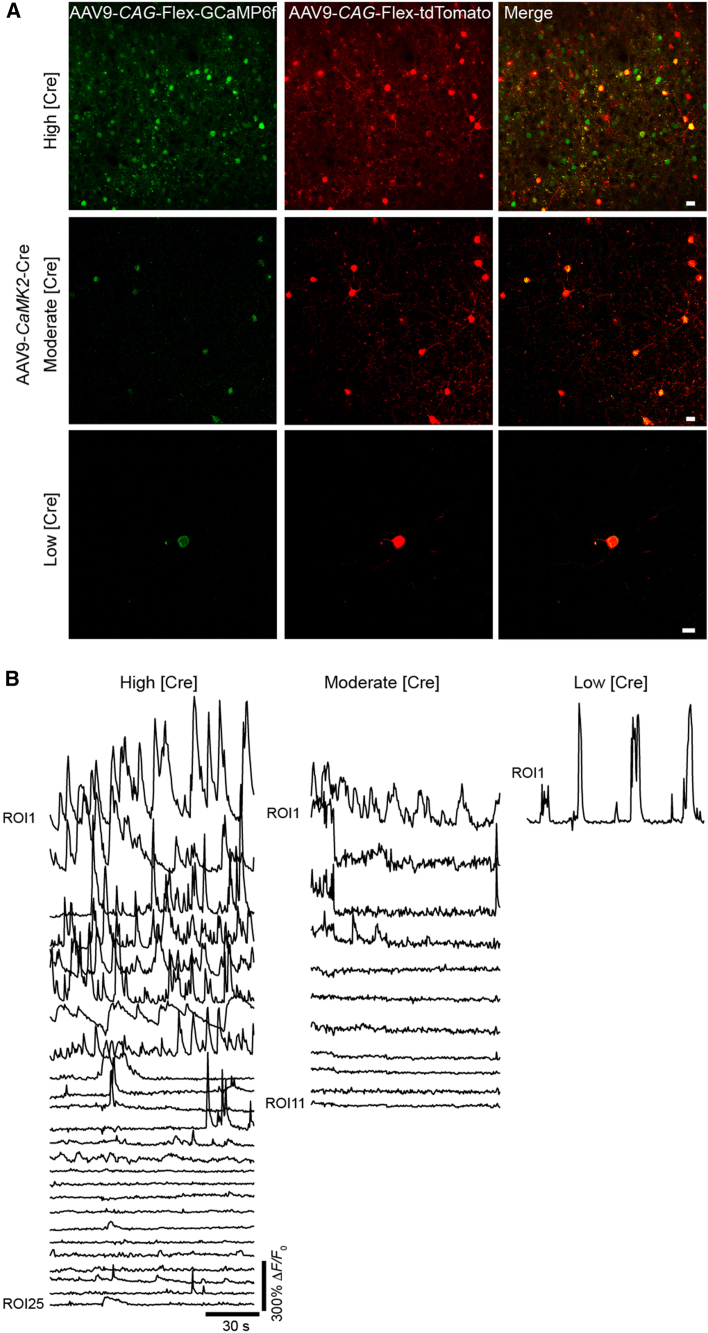
Cre concentration controls the expression of multiple Cre-dependent AAVs in individual pyramidal neurons (A and B) Two photon images (A) and GCaMP6f fluorescence traces (B) of pyramidal neurons coexpressing AAV-*CaMKII*-Cre, AAV-*CAG-*Flex-GCaMP6f, and AAV-*CAG-*Flex-tdTomato. Coexpressing cells display spontaneous calcium transients. Cre concentration regulates the density of multi-sensor expression, yet a high degree of overlap between GCaMP6f and tdTomato was consistently maintained despite Cre dilution. Scale bars, 20 μm. See also Figure S5.

**Figure 5:**
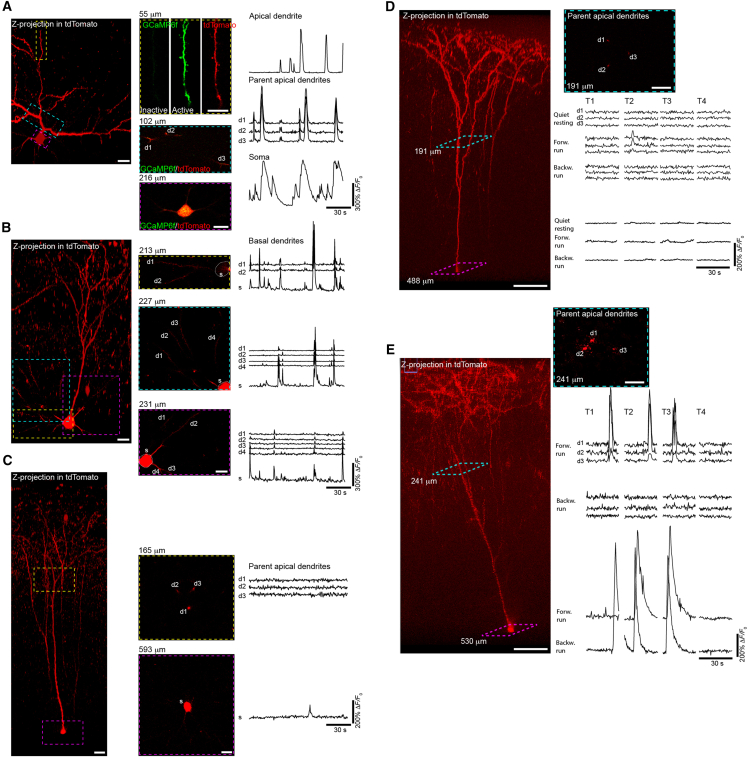
Co-expression of multiple AAVs in individual pyramidal neurons enables structure-function imaging across subcellular compartments (A) Two-photon z stack (left), 2-dimensional (2-D) imaging planes (middle; highlighted with dashed boxes in the z stack), and GCaMP6 fluorescence traces (right) of a layer 2/3 pyramidal cell in PFC coexpressing GCaMP6f and tdTomato imaged at 3 different subcellular locations: soma, apical dendritic branchpoint, and superficial dendritic branch. Apical dendrites displayed coordinated calcium activity with somatic signals. Superficial dendritic branches also displayed calcium elevation in dendritic spines and shafts. Scale bars, 20 μm except for superficial dendritic branch images (10 μm). (B) Two-photon z stack (left), 2-D imaging planes (middle; highlighted with dashed boxes in z stack), and GCaMP6 fluorescence traces (right) of a layer 2/3 pyramidal cell in PFC coexpressing GCaMP6f and tdTomato imaged at 3 different subcellular locations to capture the relationship between soma and basal dendrites. Note the coordinated activity between these two compartments. Scale bars, 20 μm. (C) Two-photon z stack (left), 2-D imaging planes (middle; highlighted with dashed boxes in z stack), and GCaMP6 fluorescence traces (right) of a layer 5 pyramidal cell in PFC coexpressing GCaMP6f and tdTomato imaged at 2 different subcellular locations to capture the relationship between soma and 3 parent apical dendrites. Somatic transient was observed without dendritic activity. Scale bars, 20 μm. (D) Two-photon z stack (left; Scale bars, 100 μm) and 2-D imaging plane (right; Scale bars, 20 μm) of single layer 5 neurons co-expressing GCaMP6f and tdTomato in awake mice running on a linear treadmill. GCaMP6 fluorescence traces show calcium activity was recorded in apical dendrites and soma during quiet wakefulness, forward running, and backward running. Localized dendritic calcium transients are detected during forward running trials without corresponding somatic activity. (E) Highly correlated dendritic and somatic activity are detected during forward running. Scale bars, 100 μm for z stack (left) and 20 μm for 2-D plane (right).

To assess whether this approach could be extended to three AAVs in a single injection, we co-injected AAV9-*CaMKII*-Cre, AAV9-*CAG*-Flex-GCaMP6f, and AAV9-*CAG*-Flex-tdTomato and found the Cre-induced recombination of both sensors. By adjusting the amount of AAV-Cre, we were able to modulate the density of neurons co-expressing GCaMP and tdTomato, ranging from sparse to high-density labeling (Figures 4A and S5). The calcium response properties were similar to those recorded under the single-AAV approach in Cre-transgenic mice (Figures 3 vs. 4B). AAVs combined with ultralow concentrations of AAV-*CaMKII*-Cre (1:100,000 dilution from original titer ≥1×10^13^ vg/mL) enabled sparse labeling of individual neurons in different cortical layers (layer 2/3 and layer 5) in the PFC (Figures 5A–5C). Z stack collections over single cells enabled the tracking of apical dendrites to cell bodies (Figure 5, left images). Calcium imaging at dendritic regions of interest and the soma revealed coordinated calcium activity between the two compartments. In thin dendrites of L5 pyramidal neurons, GCaMP and tdTomato expression was sufficient to allow calcium transient detection in spine heads and branches (Figure 5A).

To assess whether this labeling approach is suitable for experiments involving real-time imaging during behavior, we performed two-photon imaging in animals running at a fixed speed on a linear treadmill.^39^ In layer 5 neurons expressing GCaMP6f/tdTomato, we were able to track structural features from superficial apical tuft dendrites down to their corresponding somas while simultaneously recording calcium responses under conditions of quiet wakefulness and forward and backward running. In Figure 5D, we observed localized dendritic calcium transients during forward running without corresponding somatic activity. In contrast, Figure 5E shows highly correlated dendritic and somatic activity during forward running. Interestingly, the same soma was also active during backward running, though its dendrites were not. These observations suggest that the pup-injection approach supports robust labeling for simultaneous dendritic and somatic imaging at single-cell resolution, enabling the investigation of both spontaneous and behaviorally evoked neural activity.

**Figure 6:**
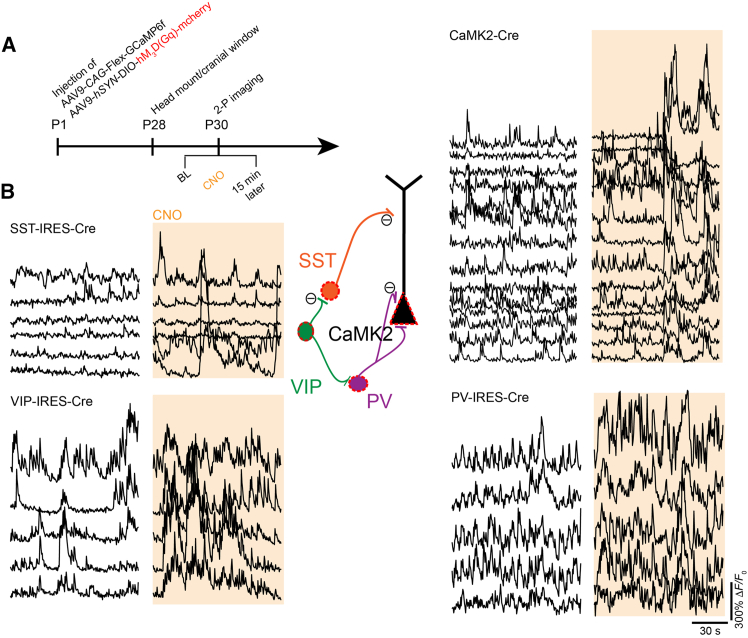
Chemogenetic modulation of distinct cortical cell types in the prefrontal cortex (A) Timeline of the DREADD-induced modulation of neuronal activity experiment. Transgenic Cre mice or AAV9-*CaMKII*-Cre were coinjected with both Cre-dependent GCaMP6f and DREADD-hM3D(Gq) as pups at P1, followed by preparation for two-photon imaging at P28. (B) On the day of imaging, layer 2/3 neurons of the PFC were recorded under wakefulness and after CNO injection (orange shaded area). CNO induced the spontaneous activation of these neuronal cell types.

We also co-injected AAV9-*CAG*-Flex-GCaMP6f and AAV9-*hSYN*-DIO-hM3D(Gq)-mCherry to assess whether DREADD activation could modulate spontaneous neuronal activity *in vivo* (Figure 6A). The hM3D(Gq) DREADD receptor engages Gq-coupled signaling pathways, leading to membrane depolarization and increased neuronal firing through multiple downstream mechanisms. To test this, we expressed these AAVs in combination with AAV9-*CaMKII*-Cre to target excitatory pyramidal neurons (3 AAVs total) and a separate combination targeting GABAergic interneurons (2 AAVs), enabling the functional interrogation of multiple cellular components within cortical microcircuits. After imaging spontaneous activity during wakefulness, the intraperitoneal injection of the inert DREADD ligand clozapine-N-oxide (CNO) produced a robust increase in spontaneous calcium signals across diverse cortical cell types (Figure 6B).

### Neonatal pup injection enables the co-expression of four adeno-associated virus constructs within the same neuronal populations of local cortical microcircuits

**Figure 7:**
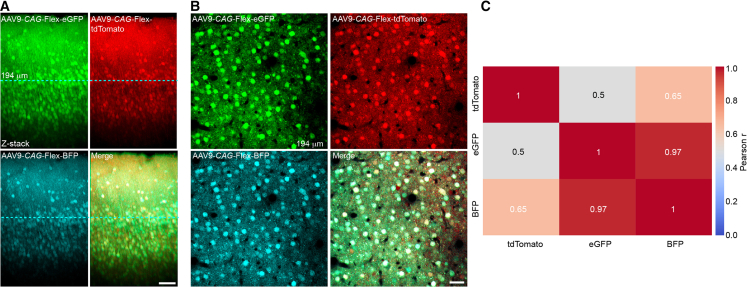
Neonatal pup injection enables the co-expression of four AAV constructs within the same neuronal populations of local cortical microcircuits (A) Two-photon z stack of the prefrontal cortex shows the expression of Cre-dependent reporters (tdTomato, eGFP, and BFP) under control of AAV9-*CaMKII*-Cre. Scale bars, 50 μm. (B) A representative layer 2/3 imaging plane from the stack at 194 μm depth reveals high overlap of fluorescent signals across pyramidal neurons (*n* = 38). Scale bars, 50 μm. (C) Pearson correlation coefficients calculated between tdTomato, eGFP, and BFP fluorescence intensities across individual cells from (B). The heatmap shows the degree of co-expression between each pair of reporters, with warmer colors indicating stronger positive correlations. Notably, eGFP and BFP exhibited a high degree of correlation (r = 0.97), suggesting strong co-expression, while tdTomato showed moderate correlation with BFP (r = 0.65) and lower correlation with eGFP (r = 0.50), indicating some variability in expression levels.

To evaluate the efficiency of neonatal pup injection for multiplexed gene expression, we co-injected four AAV constructs into the PFC and assessed their co-expression within local cortical microcircuits. Three of the constructs—encoding tdTomato, eGFP, and BFP—were Cre-dependent reporters driven by AAV9-*CaMKII*-Cre to selectively target excitatory pyramidal neurons. Two-photon imaging revealed robust and widespread expression of all three fluorophores within a single z stack, acquired from the prefrontal cortex of an adult mouse (Figure 7A). A representative imaging plane from layer 2/3 at a depth of 194 μm demonstrated clear co-expression among the fluorophores, indicating the successful co-transduction of individual neurons (Figure 7B, *n* = 38 cells; avg. cellular fluorescence intensity: tdTomato, 1722 ± 243, eGFP, 2139 ± 327, BFP 1509 ± 214). Pearson correlation coefficients for tdTomato, eGFP, and BFP fluorescence in these cells revealed strong correlations among all three reporters, with eGFP and BFP showing the highest correlation (r = 0.97), and tdTomato also correlating well with BFP (r = 0.65) and eGFP (r = 0.50). These results highlight the utility of neonatal AAV delivery for achieving reliable, multi-vector expression in the same neuronal populations.

### Neonatal adeno-associated virus-injected pups enable calcium imaging of large neuronal populations in awake, behaving mice

**Figure 8:**
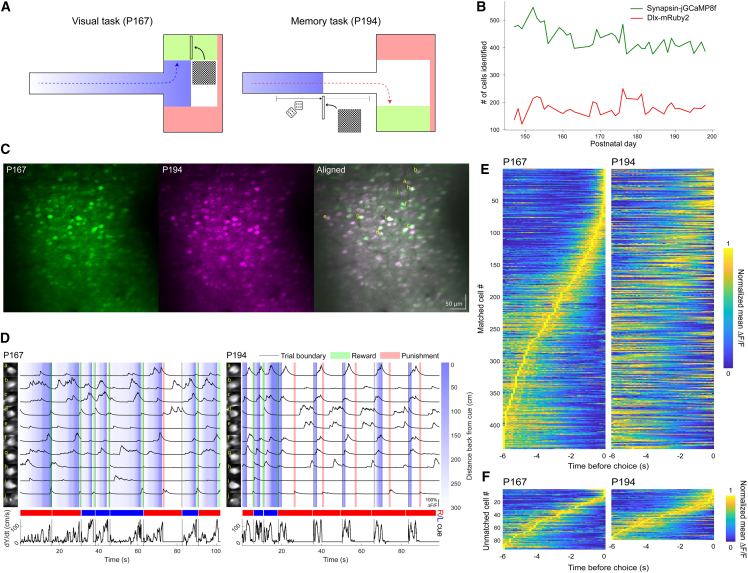
A large PPC neuronal population in a neonatal-AAV-injected mouse stably expresses jGCaMP8f over 4 weeks and exhibits activity that reflects experimental variables (A) Visual (top) and memory (bottom) cue-guided choice tasks. Shading of maze regions and colors of arrows showing correct trajectories for left and right cue trials correspond to (D). (B) Detected jGCaMP8f- and mRuby2-expressing cells in 3 imaging planes across days. (C) Mean projections of layer 2/3 neurons in PPC expressing jGCaMP8f in one imaging plane from sessions on postnatal days 167 and 194. Right: Merged projections after nonrigid alignment. The 10 cells with the highest mean Δ*F/F*_*0*_ are lettered a-j. Scale bars, 50 μm. (D) Example jGCaMP8f traces from cells with the highest mean Δ*F/F*_*0*_ during task training. Left column: mean projection of jGCaMP8f activity in a 25-pixel square around each cell during the session on P167 (left) or P194 (right). Upper panels: denoised Δ*F/F*_*0*_ traces during 8 trials in which the mouse took an efficient path to traverse the stem and made a left or right turn. Shading shows pre-cue distance and reward (sugar water) and punishment (screen flash) periods, corresponding to (A). Colored bars later in discussion traces show whether the cue and correct decision were right (red) or left (blue) for each trial. The bottom panels display the velocity along the Y axis, which is aligned with the stem. (E) Mean Δ*F/F*_*0*_ in bins of time before the choice time for cells across all 3 imaging planes that were matched between P167 and P194. The Δ*F/F*_*0*_ scale is normalized to a range of 0–1 for each cell. Cells are sorted according to the time bin with the highest mean Δ*F/F*_*0*_ on P167. (F) Same as (E) but showing cells unique to P167 and P194. In each plot, cells are sorted according to the time bin with the highest mean Δ*F/F*_*0*_ on the corresponding day.

We next explored whether neonatal AAV injection could be utilized to understand the neuronal activity of larger local networks engaged in a cognitive task. Neonatal pups were coinjected with AAV9-*hSYN*-jGCaMP8f and the GABAergic neuronal nuclear label AAV9-*mDlx*-NLS-mRuby2 in the mouse parietal cortex (Figure 8). At postnatal day 30–70, mice underwent the sequential installation of a head holder bracket followed by a cranial window (see STAR Methods). Immediately following cranial window surgery, mice were assessed for sensor co-expression. 84% of mice had strong expression of both indicators, owing to the visible feedback at the pup-injection stage. This screening step is critical for two reasons: (1) it gives novice experimenters immediate feedback on injection success, and (2) it ensures that only mice with the robust expression proceed to training, saving time and effort for animals with inadequate expression.

Following surgery recovery, mice were trained in a virtual reality environment on a visual and memory-guided cue task, progressing to the memory task after achieving >80% accuracy in the visual task (Figure 8A, see STAR Methods). Layer 2/3 neurons in the posterior parietal cortex (PPC) of a neonatal-AAV-injected mouse exhibited stable expression of jGCaMP8f and mRuby2 over 7 weeks (Figure 8B) and showed activity patterns that reflect task-specific variables (Figures 8C and 8D). Mean fluorescence projections from postnatal days 167 and 194 show consistent neuronal labeling, and nonrigid alignment confirms spatial stability across sessions (Figure 8C). Example traces from the ten most active neurons reveal reliable and trial-specific calcium dynamics aligned to behavioral events, including cue presentation, decision-making, and reward/punishment delivery (Figure 8D). Analysis of matched neurons across both sessions shows conserved temporal activity profiles sorted by peak response timing, while additional plots highlight distinct activation patterns in neurons unique to each session (Figures 8E and 8F). These findings illustrate the utility of neonatal AAV injection for long-term, high-quality calcium imaging of more extensive neuronal networks in awake, behaving animals.

## Discussion

Understanding the mechanisms underlying central nervous system function requires precise, cell-type-specific tools for visualizing and manipulating neurons in the intact brain. Here, we present a fast, efficient, and robust method for neonatal AAV delivery that enables reliable and high-level multisensor expression in cortical neurons, suitable for long-term *in vivo* imaging and functional studies. This approach significantly simplifies the technical barrier associated with adult AAV injections, offering both logistical and biological advantages.

A major strength of the neonatal AAV injection method is its dramatic improvement in throughput—once mastered, the technique enables a 40- to 50-fold increase in injection speed per animal, with high consistency across litters (Figure 1). This was a 5-fold reduction in injection time for a litter from a prior report targeting olfactory bulb regions.^40^ By avoiding the need for general anesthesia, survival surgery, and recovery monitoring required in adult injections, this approach is readily adoptable by new trainees, making it ideal for scaling up experiments and training early-stage researchers. We observed robust and tunable expression across cortical layers (layer 1 to layer 5), with expression density adjustable via AAV concentration and injection volume (Figure 4). This flexibility enables both sparse labeling for morphological analysis and dense labeling for population imaging across diverse cell types—capabilities typically limited to transgenic models. Overall, our AAV injection approach yields more region-specific reporter labeling compared to intravenous delivery into venous sinuses (transverse or retro-orbital), where labeling is typically more widespread and density is more difficult to control.^21^^,^^23^^,^^24^^,^^25^^,^^26^^,^^27^

Using this approach, we performed two-photon calcium imaging in awake animals engaged in behavioral tasks, including linear treadmill locomotion and a virtual reality–based decision-making paradigm. In layer 5 neurons co-expressing GCaMP6f and tdTomato, we tracked structural and functional features from apical dendrites to somas and observed both compartmentalized and highly correlated calcium signals depending on behavioral context (Figures 5D and 5E). These findings highlight the utility of this method for investigating the state-dependent nature of dendritic-somatic decoupling.^39^^,^^41^ At the population level, we found that layer 2/3 neurons in the posterior parietal cortex expressed jGCaMP8f stably over multiple weeks (Figure 8B) and exhibited trial-aligned calcium dynamics during task engagement. Longitudinal tracking revealed temporally conserved activity in matched neurons and distinct activation profiles in session-unique cells (Figures 8C–8F). Together, these results demonstrate that neonatal AAV injection supports high-resolution, long-term imaging of both dendritic activity and large-scale ensemble dynamics, enabling mechanistic insight into how cortical circuits encode behavior over time.

We also demonstrate that this method supports the co-expression of up to four AAVs (testing both AAV1 and AAV9), including those encoding structural markers (tdTomato, eGFP, BFP), activity sensors (GCaMP), immediate-early genes (cFos), and chemogenetic tools (DREADDs). Although we have not tested the co-expression of 5 or more AAVs in single cells, our data suggest that this approach may be feasible, albeit with the potential for inter-vector competition. Future studies could explore capsid engineering or vector optimization to increase multiplexing efficiency.

Adult animals injected as neonates exhibited no gross anatomical signs of injection and reduced neuroinflammatory markers compared to adult-injected counterparts (Figure 2). This attenuation of astrocytic activation and inflammatory signaling may reflect a developmentally permissive immune environment in neonates or an early window during which the brain is more tolerant of AAV-mediated gene delivery. Given that the injection method, electrode, and AAV serotype were identical across all age groups, the observed difference likely reflects a genuine reduction in AAV-induced immune responses following a standard recovery period (1–2 weeks for adult AAV injections). This result highlights an additional benefit of neonatal AAV delivery when long-term functional imaging or minimal perturbation of circuit homeostasis is required.

### Limitations of the study

Despite these advantages, there are notable limitations. The current implementation of the technique uses a freehand injection approach, which may introduce variability in targeting. Nonetheless, we consistently observed comparable labeling density across animals and litters, especially when using cranial windows for assessment. Robust expression of sensors and reporters was observed across 3 mm cranial windows, provided that the AAV injectates included adequate concentrations of *CaMKII*-Cre or *hSYN*-based AAVs. For investigators requiring finer spatial precision, particularly in relation to head and body positioning, a stereotaxic neonatal frame^42^ may be employed, as described and implemented in previous studies.^43^^,^^44^^,^^45^ Another limitation is the method’s incompatibility with simultaneous cranial window implantation, which requires temporal separation between viral delivery and optical access. Finally, this technique is poorly suited for subcortical targeting, where precise depth control is critical.

## Resource availability

### Lead contact

For further information please contact Joseph Cichon (joseph.cichon@pennmedicine.upenn.edu).

### Materials availability

This study did not generate new unique reagents.

### Data and code availability

• Data: Two-photon microscopy and behavioral datasets have been deposited at Zenodo and are publicly available at the date of publication. The DOI is listed in the key resources table.
• Code: All original code used in Figure 8 for data processing and analysis has been deposited at Zenodo and is publicly available at the date of publication. The DOIs are listed in the key resources table.
• All other items: Additional raw and processed datasets supporting the findings of this article will be shared by the lead contact upon request.

## Acknowledgments

We thank Marie Fina for help with animal breeding and management, Xiaoyan Zhang for technical assistance, and Jennifer Luo for help with acquiring and processing chronic imaging data. The 3D printed spherical treadmill base is courtesy of the University of Pennsylvania Libraries’ Holman Biotech Commons.

Work was supported by the 10.13039/100000002National Institutes of Health (NIH) R35GM151160-01 and American Society of Regional Anesthesia Chronic Pain Medicine Research grant to J.C., 10.13039/100000002NIH T32GM112596-09 to A.Z.W., 10.13039/100000002NIH T32NS105607 and the Blavatnik Family Fellowship in Biomedical Research to E.B.B., 10.13039/100000002NIH R01GM151556 to A.P., the 10.13039/100018115Della Martin Foundation to L.L., and the 10.13039/100000011Howard Hughes Medical Institute to L.L.

## Author contributions

Conceptualization and project administration, J.C.; methodology and investigation, S.W., E.B.B., K.B., A.Z.W., L.L., and J.C.; software, E.B.B.; writing—original draft, S.W., E.B.B., K.B., and J.C.; writing—review and editing, all authors; funding acquisition, E.B.B., A.Z.W., L.L., A.P., and J.C.; supervision and resources, L.L., A.P., and J.C.

## Declaration of interests

The authors declare no competing interests.

## Declaration of generative AI and AI-assisted technologies in the writing process

None were used in this work.

## STAR★Methods

### Key resources table

**Table:** 

REAGENT OR RESOURCE	SOURCE	IDENTIFIER
**Antibodies**		

Chicken Polyclonal anti-GFAP	Abcam	Cat# ab4674; RRID: AB_304558
Rabbit Monoclonal anti-Iba1	Abcam	Cat# ab178846; RRID: AB_2636859
Goat anti-Rabbit IgG (H+L) Cross-Adsorbed Secondary Antibody, Alexa Fluor 594	ThermoFisher	Cat# A-11012; RRID: AB_2534079
Goat anti-Chicken IgY (H+L) Cross-Adsorbed Secondary Antibody, Alexa Fluor Plus 647	ThermoFisher	Cat# A32933; RRID: AB_2762845

**Bacterial and virus strains**		

pAAV9-mDlx-NLS-mRuby2	Addgene^46^	Cat# 99130-AAV9; RRID: Addgene_99130
pGP-AAV9-syn-jGCaMP8f-WPRE	Addgene^47^	Cat# 162376-AAV9; RRID: Addgene_162376
pAAV9.CAG.Flex.GCaMP6f.WPRE.SV40	Addgene^48^	Cat# 100835-AAV9; RRID: Addgene_100835
pAAV9-Flex-tdTomato	Addgene	Cat# 28306-AAV9; RRID: Addgene_28306
pZac2.1-GfaABC1D-lck-jGCaMP8f	Addgene	Cat# 176759-AAV9; RRID: Addgene_176759
pAAV9-hSyn-DIO-hM3D(Gq)-mCherry	Addgene^49^	Cat# 44361-AAV9; RRID: Addgene_44361
pENN.AAV9.CamKII 0.4.Cre.SV40	Addgene	Cat# 105558-AAV9; RRID: Addgene_105558
pAAV9-CAG-FLEX-EGFP	Addgene	Cat# 59331-AAV9; RRID: Addgene_59331
pAAV9-CAG-FLEX-BFP	Vector biolabs	Lot# 250331#49
pAAV.Syn.GCaMP6f.WPRE.SV40	Addgene^48^	Cat# 100837-AAV9; RRID: Addgene_100837
AAV9-hSYN1-DIO-mFOS.eGFP-WPRE	Vector biolabs	Lot# 230522#59

**Chemicals, peptides, and recombinant proteins**		

Fast Green FCF	Sigma-Aldrich	F7252

**Deposited data**		

Calcium imaging and behavioral data	Zenodo	https://doi.org/10.5281/zenodo.17117612

**Experimental models: Organisms/strains**		

C57BL/6	Jackson Laboratory	000664
Rbp4-Cre	GENSAT project at Rockefeller University	031125
PV-IRES-Cre	Jackson Laboratory	008069
Sst-IRES-Cre	Jackson Laboratory	013044
VIP-IRES-Cre	Jackson Laboratory	010908

**Software and algorithms**		

Prism version 9.3.1	GraphPad	N/A
Fiji version 2.9.0	NIH	https://imagej.net/software/fiji/
ImageJ version 2.1.0	NIH	https://imagej.net/ij/index.html
ANY-Maze version 7.0	Stoelting Co.	https://www.any-maze.com/
R version 4.3.2	R Core Team	https://www.R-project.org.
ARTool R package version 0.11.2	CRAN	https://github.com/mjskay/ARTool/
Harvey Lab Mouse VR	Harvey Lab and HMS Research Instrumentation Core, Harvard Medical School^50^	https://github.com/HarveyLab/mouseVR
MATLAB version R2024a	MathWorks	https://www.mathworks.com/products/matlab.html
ViRMEn version 12-Feb-2016	Tank Lab, Princeton University^51^	N/A
Python version 3.11	Python Software Foundation; conda-forge	https://anaconda.org/conda-forge/python
CaImAn version 1.11.4-dev.wisserblackwood2025	Chklovskii Lab, Flatiron Institute, Simons Foundation^52^; this paper	https://doi.org/10.5281/zenodo.17114547
Mesmerize-core version 0.4.0-dev.wisserblackwood2025	Giovannucci Lab, UNC/NCSU; this paper	https://doi.org/10.5281/zenodo.17117510
Custom Python and MATLAB code for Figure 8	This paper	https://doi.org/10.5281/zenodo.17117568

**Other**		

Glass micropipettes	Drummond	5-000-1001-X10

### Experimental model and study participant details

#### Animals

Mice (C57BL/6 strain background; Postnatal (P) day 1-3 for AAV injections and P30-190 for *in vivo* imaging) of both sexes were maintained at the University of Pennsylvania (Penn) Perelman School of Medicine John Morgan animal facility and were housed under standard conditions (12-h light-dark cycle [lights on at 7:00 a.m.]) with controlled temperature and humidity conditions and access to food and water *ad libitum*. All animal handling was in accordance with guidelines set forth by the School of Medicine’s Institutional Animal Care and Use Committee (approved protocols no. 807237 and 803844 for Penn).

### Method details

#### Injection strategies

The genetically encoded calcium indicators (GECIs) GCaMP6f and jGCaMP8f were used for calcium imaging of pyramidal neurons and interneurons in the cortex. GECI expression was achieved using intracranial AAV injections of neonates. A glass micropipette (Drummond, 5-000-1001-X10) was pulled and beveled. A plunger was lightly oiled and inserted into the micropipette to pull the AAV mixture. Subsequently, pups at postnatal day 0-2 were anesthetized by hypothermia until immobile (typically ∼3-5 min on ice), and the micropipette was used (freehand) to penetrate the skin and skull and deliver ∼100-500 nL of the virus mixture (Addgene AAV with typical titer ≥ 7×10^12^ vg/mL unless otherwise noted). Glass micropipettes were used for their 1 μL graduations, allowing experimenters to approximate the volume drawn into the electrode and estimate the volume delivered. Larger volumes and multiple injections can be performed, but these approaches are not included in the present work. Cortical injection sites were determined using landmarks—skull suture lines and head veins—as reference points (Figure 1B). Imaging was performed in male and female mice aged 1 to 6 months, following a minimum of 4 weeks of AAV expression; however, earlier imaging is possible if warranted. Mice were group-housed in temperature- and humidity-controlled rooms on a 12-hour light/dark cycle after injections.

In Figure 2, pup-injected and adult-injected GCaMP6f expression patterns were compared within the same animals. Mice that had been injected with AAV9-*hSYN*-GCaMP6f (Addgene, 100837)^48^ at P1 were anesthetized at P30-40 and re-injected with the same virus in the contralateral medial prefrontal cortex (mPFC; +0.5–1.0 mm anterior of bregma and 0.3–0.5 mm lateral to midline). For the adult injection, a total of 0.1–0.2 μl of AAV virus was diluted 5 times and injected (Picospritzer III; 15 p.s.i., 12 ms, 0.8 Hz) at a depth of 300-500 μm over 10–15 min using a glass microelectrode.^39^ The electrode was kept in place for 10 min before retracting.

In Figure 3, AAV9-*CAG*-FLEX-GCaMP6f (Addgene, 100835; 100 nL of AAV per mouse)^48^ targeted to the neonatal prefrontal cortex (∼0.5–1 mm anterior to bregma, 0.5 mm lateral to midline) induced GCaMP6f expression in Rbp4-Cre (GENSAT project at Rockefeller University, 031125), PV-IRES-Cre (Jackson Laboratory, 008069), Sst-IRES-Cre (Jackson Laboratory, 013044), and VIP-IRES-Cre (Jackson Laboratory, 010908) mice. Cre-expressing mice were genotyped for the presence of Cre based on established protocols as adults.

In Figures 4, 5, and S5, pyramidal neurons were labeled using recombinant AAV9-*CaMK2*-Cre (Addgene, 105558), AAV9-*CAG*-FLEX-tdTomato (Addgene, 28306), AAV9-*CAG*-FLEX-GCaMP6f (Addgene, 100835),^48^ and/or AAV9-*hSYN*-DIO-mFos-eGFP-WPRE (Vector Biolabs, 1.68 x 10^13^ GC/mL, Lot# 230522#59). Different labeling densities were accomplished by diluting Cre virus in artificial cerebrospinal fluid (aCSF)/fast-green dye (Sigma Aldrich, F725) solution followed by mixing with GCaMP6f and tdTomato viruses. The lowest Cre concentration injection consisted of a final AAV mix containing 80% GCaMP virus, ∼17.5% tdTomato virus, and ∼2.5% Cre virus/fast-green dye.

In Figure 6, DREADD-induced modulation of pyramidal neurons was accomplished with expression of Cre-dependent DREADD-hM_3_D(G_q_) (AAV9-*hSYN*-DIO-hM_3_D(G_q_)-mCherry; Addgene, 44361)^49^ under the human *synapsin-1* promoter in Cre-positive mice or mice coexpressing AAV9-*CaMK2*-Cre (Addgene, 105558). Pyramidal neuron activity was imaged before and ∼15-20 minutes after CNO i.p. injection (Tocris, 4936; 3 mg/kg; solution of CNO in saline 0.3 mg/ml).

In Figure 7, AAV9-*CaMK2*-Cre (Addgene, 105558), AAV9-*CAG*-FLEX-tdTomato (Addgene, 28306), AAV1-*CAG*-FLEX-eGFP (Addgene, 59331), and AAV9-*CAG*-FLEX-BFP (Vector Biolabs; 1.06 x 10^13^ GC/mL) were targeted to the neonatal prefrontal cortex (∼0.5–1 mm anterior to bregma, 0.5 mm lateral to midline).

In Figure 8, AAV9-*hSYN*-jGCaMP8f (Addgene, 162376, titer ≥ 1×10^13^ vg/mL)^47^ and AAV9-mDlx-NLS-mRuby2 (Addgene, 99130, titer ≥ 1×10^13^ vg/mL)^46^ were injected into the left parietal cortex (midway between bregma and lambda, ∼1 mm lateral to midline) of neonatal mice. AAV-mDlx-NLS-mRuby2 labeling of interneuron nuclei served as a structural marker to aid in image registration across imaging sessions. The injected AAV mixture contained 33% jGCaMP8f virus, 33% mRuby2 virus, and 34% diluted fast-green dye.

#### Behavioral testing

In Figure S2, non-injected and pup-injected mice were assessed using the marble burying test (MBT), rotarod performance, and the tail suspension test (TST). In the MBT, mice were placed in a large rat cage with ample bedding and 20 marbles evenly distributed across the surface. After 30 minutes, the number of marbles buried (defined as ≥ two-thirds covered by bedding) was recorded. For the rotarod test, the AccurRotor EzRod system was used. The rod accelerated from 1 to 45 RPM over 80 seconds, with each trial lasting 180 seconds. The maximum RPM achieved was used as a proxy for motor performance. In the TST, mice were then suspended by their tails using ∼15 cm of strong adhesive tape affixed to the top of the testing apparatus. Although the total test duration was 6 minutes, only the final 4 minutes were analyzed for periods of mobility, using ANY-maze software.

#### Surgical preparation before *in vivo* imaging in adult animals

In Figures 3, 4, 5, 6, and 7, mice underwent a surgical procedure to attach a head holder mount and create an imaging window for two-photon microscopy. In brief, mice were anesthetized with a mixture of 100% oxygen at 2 L/min and 1-4% isoflurane or ketamine-xylazine (100 mg/kg, 10 mg/kg). A heating pad was used to maintain the animal’s body temperature at approximately 37°C. The mouse’s head was shaved, and its skull surface was exposed with a midline scalp incision. The periosteal tissue over the skull surface was removed without damaging the temporal and occipital muscles. A head holder consisting of two parallel metal bars was attached to the animal’s skull. In Cre-positive transgenic mice injected with AAV, <1% of mice were negative for GCaMP fluorescence, suggesting that genotyping error or off-target injection was a rare event. In positive mice, a small skull region (∼2-4 mm in diameter) located over the interfrontal suture was removed, and a round glass coverslip (approximately the same size as the bone being removed) was affixed to the skull with Loctite 495 followed by dental acrylic cement. This window enabled imaging of the medial PFC (+0.5–1.0 mm anterior of bregma and 0.3–0.5 mm lateral to midline) or the primary motor cortex (M1; +1.5mm, +1.5mm). Upon recovering from surgical anesthesia, mice with head mounts were habituated daily (two sessions of 30 minutes with a 15-minute break) starting on postoperative day 1 in a custom-built body support to minimize potential stress effects of head restraining and imaging. Mice tolerated surgery and stress related to the perioperative period, as indicated by a 0–10% drop in weight. Imaging experiments were started on postoperative day 2-3 after window implantation.

In Figure 8, mice underwent a two-stage surgery: 1) attachment of a custom-designed headplate, 2) implantation of a cranial window. Mice were anesthetized with a mixture of 100% oxygen at 1 L/min and 1-4% isoflurane, and body temperature was maintained at 37 °C with a heating pad. First, the skull was placed into zygomatic ear cups (Kopf Instruments) and leveled. Following scalp and skull preparation (as described above), a custom O-shaped headplate was affixed to the skull using dental cement (Parkell Metabond and methyl methacrylate), centered over the left posterior parietal cortex (−2.0 mm AP, −1.7 mm ML). Mice were allowed to recover for 7 days. Before the second surgery, mice received an intraperitoneal injection of 0.5 mg/kg dexamethasone before the incision to reduce brain and dural inflammation. Mice were attached to the stereotaxic frame using a head-holding device (Narishige SR-9AM) attached to the headplate, rather than ear bars, to reduce brain swelling. A 3-4 mm diameter craniotomy centered above the PPC (crossing the sagittal suture) was performed. A glass cranial window consisting of two round bonded glass coverslips (5 mm and 3 mm diameter coverslips glued together by UV glue (Norland NOA71) was inserted over the exposed dura and affixed to the surrounding skull with Loctite 454 monolayer followed by careful application of dental acrylic cement over this monolayer.^53^ After recovery, typically on the same day, mice underwent two-photon imaging to verify GCaMP expression in the PPC. Subsequently, animals recovered for 2-3 weeks before starting imaging experiments to enable sufficient clearing of the optical window.

#### Two-photon imaging in adult mice

In Figures 3, 4, 5, 6, and 7, on the day of imaging, awake mice were positioned in the custom head holder device under the two-photon microscope. In Figures 3, 4, 5, and 6, *in vivo* two-photon imaging was performed with an Olympus DIY RS two-photon system (tuned to 800–1050 nm) equipped with a Coherent Discovery NX laser. Fluorescence emission was collected in a non-descanned configuration through the objective lens and directed to the detector arm via the nosepiece dichroic mirror (NDM). Emission was spectrally separated using filter cubes and a splitting dichroic mirror (SDM). With the SDM570 in place, wavelengths >570 nm were directed through Olympus F30FRCY5 to channels 1 (Cy5)/2 (Red), while wavelengths <570 nm were directed through Olympus FV30-FOPT-VG cube to channels 3 (Green)/4 (Violet). In Figure 7, BFP was excited at 800 nm, GFP/GCaMP at 920 nm, and tdTomato at 1050 nm. Emission was detected by GaAsP photomultiplier tubes (PMTs). We minimized movement-associated image artifact by head (secured metal head bars) and body (with a plastic sleeve) restraint on the imaging platform. Pyramidal neurons and interneurons in cortical regions were randomly chosen and recorded for 2-min sessions under awake conditions, except for in Figures 5D and 5E where pyramidal neurons were recorded for 30 s in running trials.^39^ All experiments were performed using a ×20 Olympus objective (XLUMPLFLN; 1.00 NA, 2.0 mm working distance) immersed in aCSF, with ×2-3 digital zoom. Images were acquired at a frame rate of 2-4 Hz (2-μs pixel dwell time). Image acquisition was performed using Olympus Fluoview software and analyzed post hoc using ImageJ software version 2.1.0.

In Figure 8, on each imaging day, awake mice were transferred onto a spherical treadmill and head-fixed under a Neurolabware standard microscope equipped with an electrically tunable lens (ETL, Optotune) for multi-plane imaging. A metal light-blocking sleeve was placed into a groove in the headplate to prevent stray light from the VR projector from interfering with imaging.^54^ At the start of each session, the femtosecond laser was tuned to 1050 nm, and the ETL was set to cycle between 3 planes spaced 30 μm apart, switching each frame (31.25 Hz). The mDlx-mRuby2 signal was recorded for 45-150 frames (15-50 frames/plane, depending on expression level) at each relative Z position of the objective from +30 to −30 μm, stepping by 5 μm. This structural stack was mean-projected across time and used to compute shifts for 3D alignment with a reference image from a previous day in two steps. First, the top plane at each Z position was cross-correlated with the reference top plane, and the best X and Y shifts were inferred from the highest cross-correlation value across all planes. Secondly, the X/Y shifted stack at each Z position was correlated with the reference image, and a linear-offset Gaussian was fit to these correlations as a function of Z. The center parameter of the Gaussian was taken as the best Z shift. The shifts were converted to μm and used to adjust the objective position. This process was repeated until the computed shifts were each < 5 μm and the mean projection at Z = 0 visually matched the reference. Once alignment was achieved, the laser was tuned to 920 nm, and training began on a behavioral task (see “behavioral training in VR”). During each trial (4.5-458 s; median = 15.8 s), jGCaMP8f signals were recorded (583 x 862 x 60 μm or 512 x 796 x 3 px; ×1.2 magnification; 10.4 vol/s).

#### Behavioral training in VR

Adult mice (2-6 months) expressing jGCaMP8f and Dlx-mRuby2 were water restricted (≥1.0 mL/day to maintain 83-85% original weight^55^) and trained on a two-alternative choice task in a virtual reality (VR) T-maze (Figure 8A). Training took place on a VR rig consisting of a spherical treadmill coupled to a parabolic-screen projection system,^50^ controlled by the ViRMEn VR engine^51^ in MATLAB. At the start of each trial, mice were “teleported” to the base of the T and the trial’s GCaMP recording started (triggered from VR computer by UDP communication). In visual trials (all trials in earlier sessions; warmup and catch trials in later sessions), a “billboard” cue was visible in the left or right arm of the T maze throughout the trial. In memory trials (non-warmup/catch trials in later sessions), the same cue was visible at the left or right side of the stem, at a distance from the junction sampled uniformly at random between 8.5 and 153.8 cm. The cue side was chosen randomly on each trial, with the side probabilities adjusted according to performance on the past 40 trials to correct side biases.^56^^,^^57^ To complete each trial correctly, mice had to use the treadmill to initiate the trial by moving in any direction (≥5.7 cm/s), proceed 219 cm to the T junction, and go 37 cm into the arm of the T on the same side as the cue. The reward for each correct response was a 2 μl drop of sugar water (10% sucrose). When mice entered the incorrect (opposite side) response area, traveled within 8.5 cm of the back wall, or did not respond within 120 seconds of trial initiation, the trial ended and a full-screen flash (10 flashes over 1 second) was presented, followed by 8 extra seconds of timeout before the next trial. After each reward or punishment, the screen was blanked, and there was a 1-1.5 second random timeout, during which time the trial’s GCaMP recording ended. Then there was a further delay until the treadmill remained still (<4.6 cm/s X/Y speed and <0.1 radians/s turning speed) for a period of 200 ms to avoid bouts of disengaged running spanning multiple trials. Finally, the screen was unblanked, and the next trial began.

Mice were generally trained for 1 hour each weekday. They progressed through the following stages of shaping and training. On day 1, mice were shaped to lick a blunt feeding needle for rewards: 2 μl drops of sugar water were released whenever a lick was detected, up to a maximum rate of 2 drops/s, until 1 mL had been released. Mice always obtained the maximum reward volume in less than 1 hour. For 1-6 days, mice were shaped to run forward in VR through a corridor the same length as the T-maze and reach the end within 120 seconds. Reward drops were dispensed at invisible thresholds, initially located every 28.5 cm forward from the starting location, and at the end of the corridor. Each time mice surpassed a criterion of 80% trials finished over the last 20 trials, the threshold spacing was increased by 28.5 cm, up to a maximum of 113.9 cm. When the criterion on the maximum spacing was surpassed, shaping ended for the day and training started the next day. For 14-16 days, mice were imaged and trained on only visual trials (cue at the end of the maze) of the two-alternative choice task. Training on this version of the task continued until a criterion of 90% accuracy was surpassed in a single day, excluding trials with no left or right choice. Additional training days were sometimes added to collect more images during learned behavior. For 23-32 days, mice were imaged and trained on memory trials of the two-alternative choice task. During memory sessions, visual trials were also used for warm-up blocks, recovery blocks triggered by <55% accuracy on the past 40 trials, and otherwise on 15% of trials to maintain motivation.^57^ Training continued until a criterion of 80% accuracy was surpassed or imaging quality deteriorated.

#### Immunostaining, imaging, and analysis of adult brains following neonatal AAV injection

For direct comparison of pup-injected and adult-injected brain hemispheres, neonatal (P1) AAV injections were performed on right hemispheres, and adult (P30) injections were performed on the left hemisphere in 4 animals. Upon brain harvest (P45) and overnight post-fix in 4% paraformaldehyde, brains were placed in PBS with 0.02% sodium azide until ready for sectioning. To preserve laterality before sectioning, a 25-gauge needle was pushed through the right side of the brain. A compresstome (Precisionary Instruments VF-510-0Z) was then used to prepare 40μm thick coronal sections for Iba1 and GFAP staining (1:4 series cut, 1 well stained). Sections were blocked in 2.5% normal goat serum in PBST (0.3% Triton X-100 in PBS) for 2 hours at room temperature, followed by a 48-hour incubation at 4°C in chicken anti-GFAP primary antibody (abcam ab4674, 1:1000) and rabbit anti-Iba1 (abcam ab178846, 1:1000) in 2.5% normal goat serum in PBST. Sections were then washed in PBS, followed by a 2-hour incubation with Alexa Fluor 594 goat anti-rabbit (ThermoFisher 1:200) for Iba1 visualization and Alexa Fluor 647 goat anti-chicken (ThermoFisher 1:200) for GFAP visualization. Following a final wash in PBS, sections were mounted and coverslipped with mounting medium containing a DAPI counterstain (Southern Biotech 1011-20). Sections were imaged on a fluorescence microscope (Keyence BZ-X810) at 10x (PlanApo, NA: 0.45) or 20x (PlanApo, NA: 0.75) using a DAPI (Ex: 360/40, Em: 460/50), GFP (Ex: 470/40, Em: 525/50), TRITC (Ex: 545/25, Em: 605/70), and Cy5 (Ex: 620/60, Em: 700/75) filter cube. Images were stitched and then processed with a haze reduction filter using the manufacturer’s provided software.

Iba1 and GFAP expression were quantified and presented as animal-level averages using two approaches: (1) fluorescence intensity measurements across AAV-expressing regions of interest (ROIs) in pup- and adult-injected brains (Figure 2E), and (2) expression indices derived from the density of Iba1- and GFAP-positive cells within each ROI (Figure 2F). For fluorescence intensity analysis, a fixed-length line was manually placed over the AAV-expressing ROI in each coronal section, and the fluorescence intensity along this line was extracted using the Plot Profile tool in ImageJ (Fiji; version 2.9.0). Expression indices were calculated by dividing the number of Iba1 or GFAP positive cells in a manually drawn ROI by the area of the ROI, multiplied by a scaling factor of 10,000. Cell counts were obtained using a particle analysis pipeline in Fiji. Briefly, each section was manually thresholded, binarized, and processed using a watershed algorithm to separate individual cells. Particle detection was then performed using minimum size thresholds of 5 pixels^2^ for Iba1 and 10 pixels^2^ for GFAP. For each animal, values from four mPFC coronal sections were averaged to generate the final fluorescence intensity and expression index measurements.

#### Analysis of *in vivo* GCaMP and reporter signals

For Figures 3, 4, 5, 6, and 7, during recordings, motion-related artifacts were typically less than 2 μm. Vertical movements were infrequent and minimized by two metal bars attached to the animal’s skull (described above) and a custom-built body support. All timelapse images from each individual field of view were motion-corrected and referenced to a single template frame using cross-correlation image alignment (TurboReg plugin for ImageJ version 2.1.0). ROIs corresponding to visually identifiable somas (pyramidal cells and interneurons) were selected manually from the field of view. Imaging planes were acquired from L2/3 and L5, corresponding to cells positioned ∼150–350 μm and ∼500–750 μm from the pial surface, respectively. Somas that could be identified in all imaging sessions were included in the dataset. All pixels inside the ROI were averaged to obtain a fluorescence trace for each ROI. Background fluorescence was calculated as the average pixel value per frame from a region without GCaMP expression (blood vessel) and subtracted from the time-series fluorescence traces. The baseline (*F*_0_) of the fluorescence trace was estimated by the average of inactive portions of the traces (∼2 s). In Figures 3, 4, 5, and 6, we did not smooth the raw fluorescence trace (raw traces are presented throughout the manuscript in each figure). The Δ*F*/*F*_*0*_ (%) was calculated as Δ*F*/*F*_0_ = (*F* − *F*_0_) / *F*_0_ × 100. Representative traces from a given cortical region were selected at random. In Figure 7, fluorescence intensities for tdTomato, eGFP, and BFP were measured from manually segmented ROIs in ImageJ (Fiji, version 2.9.0), and Pearson correlation coefficients were calculated across individual cells (n = 38) using z-scored values.

For Figure 8, calcium imaging data processing was done in Python. Movies from all trials on each imaging day were first concatenated in time and motion corrected using piecewise-rigid alignment (NoRMCorre,^58^ as implemented in the CaImAn software package), separately for each imaging plane. The motion correction results were manually verified, and correction was redone with higher maximum shifts as necessary if the initial correction was insufficient. To obtain a starting estimate of cell locations, mean projections of each corrected movie were first brightness corrected by dividing by a brightness map obtained by median-filtering the mean projection with a uniform 25x25 pixel kernel. Binary masks for putative cells were then extracted using adaptive thresholding followed by morphological operations to remove small holes and components (as implemented in CaImAn^52^). This adaptive thresholding method was applied to both jGCaMP8f and mRuby2 recordings on each day to count cells and assess the stability of fluorophore expression over 7 weeks (Figure 8B). Masks extracted from the jGCaMP8f recording were also used to seed constrained nonnegative matrix factorization (CNMF^59^), which identifies, denoises, and refines active components. CNMF was performed using the CaImAn and Mesmerize-core software packages. The components identified from each day’s concatenated movie were evaluated for signal-to-noise ratio (SNR) and spatial consistency across active frames (“r-value”). SNR was assessed using a gamma rather than Gaussian background noise distribution to handle highly skewed noise in the darkest parts of the field of view.^60^ Initially, components with SNR > 1.2 or r-value > 0.85 were accepted, and components with SNR < 0.5 or r-value < 0.1 were rejected regardless of the other metric. After this automatic evaluation, the mean and local correlation images and Δ*F*/*F*_0_ of selected components were inspected to tweak the accepted set if necessary.

Once active cells were identified from each recording, their denoised calcium traces were exported for further processing. As defined in the CNMF framework, the denoised trace is the convolution of a spike train with an AR(1) process (decaying exponential) representing a single calcium transient.^59^ The model also identifies a global background component, which is not constrained to be local in space and therefore typically captures variation in average fluorescence over time. Denoised traces were detrended by removing the 8th percentile in 500-frame moving windows. Each cell’s baseline fluorescence *F*_0_ was estimated as its residual from detrending plus the 8th percentile of the background component within the cell’s spatial mask in a 500-frame moving window. Then Δ*F*/*F*_0_ was computed as the denoised and detrended trace divided by this baseline.

To observe long-term changes in neural coding, we aligned recordings from different days and matched overlapping cells. To align two recordings, first a piecewise rigid spatial mapping was computed between the brightness-corrected mean projections of each recording using NoRMCorre,^58^ with an initial manual shift applied if necessary to allow the maximum automatic shift to be reduced. This result was used to map the cell masks from one recording onto the coordinate space of the second recording. Finally, cell masks were matched by solving the linear sum assignment problem, using binary intersection over union as the cost (set to 1 for pairs greater than 10 pixels apart) and keeping only matches with cost < 0.8. To produce Figure 8, denoised calcium traces from matched cells between two representative sessions were loaded and plotted against behavioral data captured from the VR system using custom MATLAB code.

### Quantification and statistical analysis

Animals of both sexes were randomly assigned to experimental groups, but specific comparisons were made. No statistical methods were used to predetermine sample sizes, but our sample sizes for *in vivo* imaging and behavior studies are similar to those reported in our previous publications and others.^37^^,^^38^^,^^61^^,^^62^^,^^63^ We tested the data for normality using the Shapiro-Wilk test and performed parametric statistical tests. If normality was not present, we performed nonparametric tests, including the Wilcoxon rank sum test (instead of a t-test) to compare two groups, Kruskal-Wallis (instead of a one-way ANOVA) to compare more than two groups, and aligned rank transform (ART) ANOVA (instead of a 2-way repeated measures ANOVA) to compare 2 factors. Tests were computed in GraphPad Prism version 9.3.1., or with the R package ARTool (version 0.11.2). Exact *P* values are reported in figures and legends.
