# Supplementary material for: Rapid neonatal AAV delivery for adult cortical two-photon imaging of genetically encoded sensors
Source: iScience. 2025 Oct 30;28(11):113898. doi: 10.1016/j.isci.2025.113898 (PMC12661347; doi:10.1016/j.isci.2025.113898)
Supplement: Document S1. Figures S1–S5 [file mmc1.pdf]

iScience, Volume *28*

Supplemental information

Rapid neonatal AAV delivery for adult cortical two-photon imaging of genetically encoded sensors

Stephen Wisser, Ethan B. Blackwood, Kallol Bera, Andrzej Z. Wasilczuk, Loren L. Looger, Alex Proekt, and Joseph Cichon

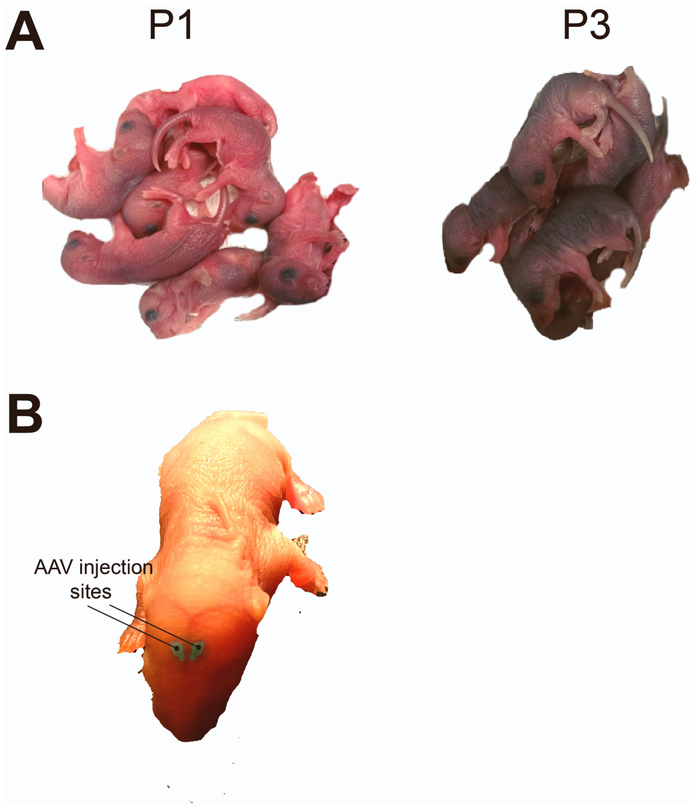

Figure S1. Postnatal age increases skin pigmentation, related to Figure 1
(A) Photographs comparing postnatal day 1 (P1) and P3 pups highlight significant skin pigmentation changes. Increased skin darkening by P3 obscures anatomical landmarks, making accurate injection targeting more challenging.
(B) Photograph of a P1 neonatal mouse following bilateral injection of an AAV/fast green mixture, demonstrating symmetrical targeting and distribution of the injectate.

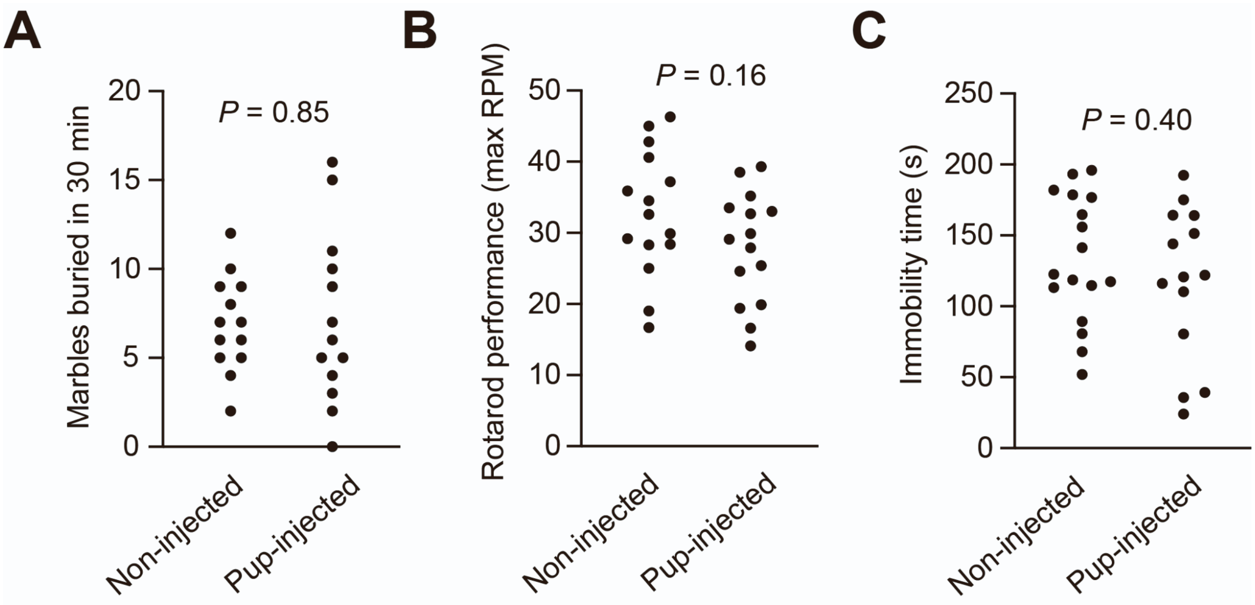

Figure S2. Neonatal AAV injections do not alter animal behaviors in adulthood, related to Figure 2
Comparison of non-injected and pup-injected mice in the marble burying test (A), rotarod performance (B), and tail suspension test (C), conducted between postnatal days 30 and 40. No significant behavioral differences were observed between the two groups. Exact *P*-values shown (Mann-Whitney test).

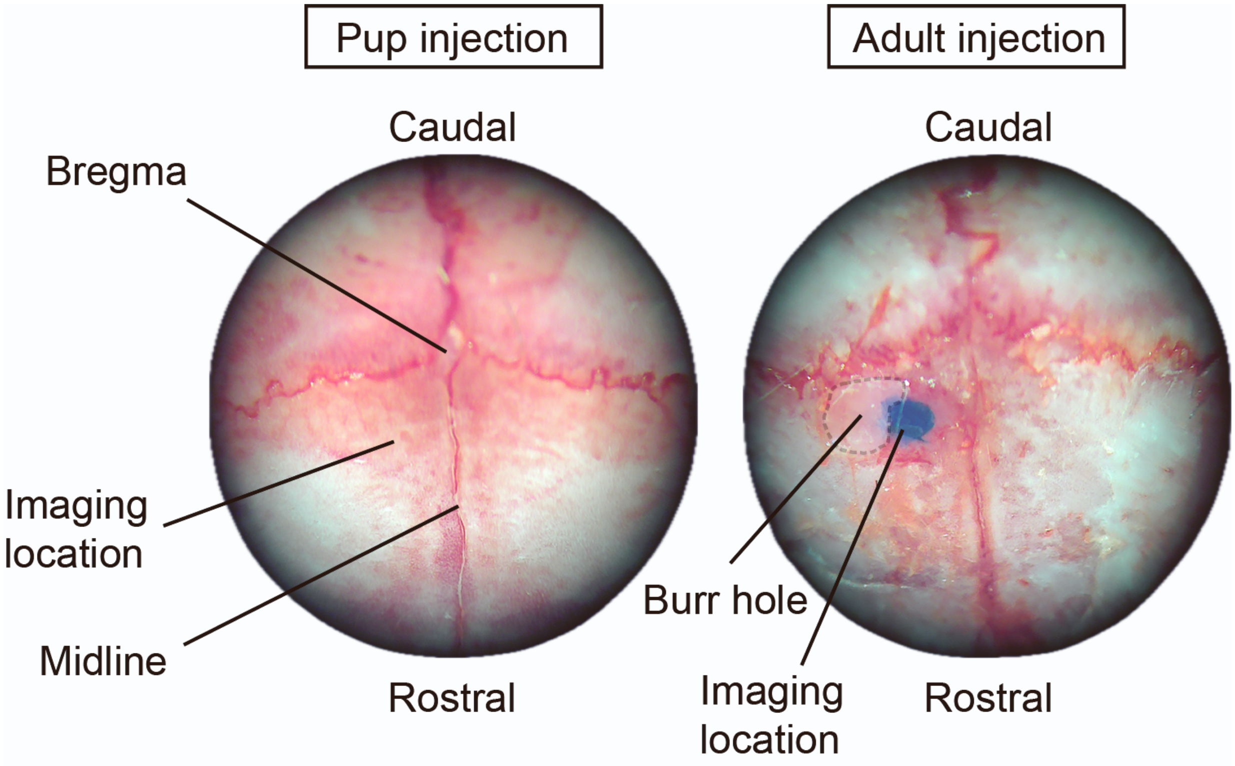

Figure S3. Skull fully recovers following neonatal AAV injection, related to Figure 2
Photographs of adult mouse skulls following neonatal versus adult AAV injections. Note that skulls from adult-injected mice exhibit lasting deformation at the injection site, whereas those injected as pups show no visible signs of prior injection.

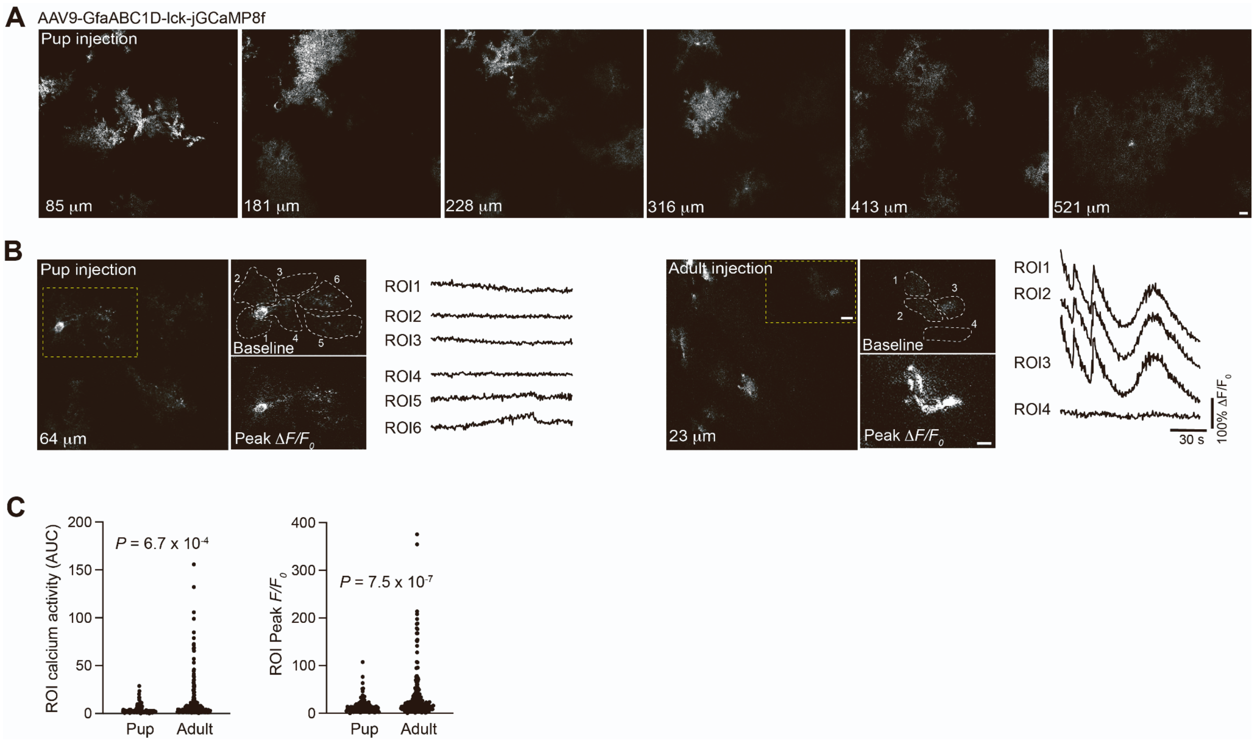

Figure S4. Neonatal AAV injection enables calcium recording in glial cells, related to Figure 2
(A) Representative two-photon images of astrocytes expressing jGCaMP8f across the PFC cortical column. Scale bar, 20 μm.
(B) Two-photon images (left) and jGCaMP8f fluorescence traces (right) from mice injected with AAV9-*GfaABC1D*-lck-jGCaMP8f either neonatally or in adulthood. Scale bar, 20 μm.
(C) Adult-injected mice (n = 203 ROIs from 3 mice) exhibited altered astrocytic morphology and displayed more frequent and larger spontaneous calcium events compared to neonatally injected mice (n = 107 ROIs from 3 mice). Exact P-values shown (Mann-Whitney test).

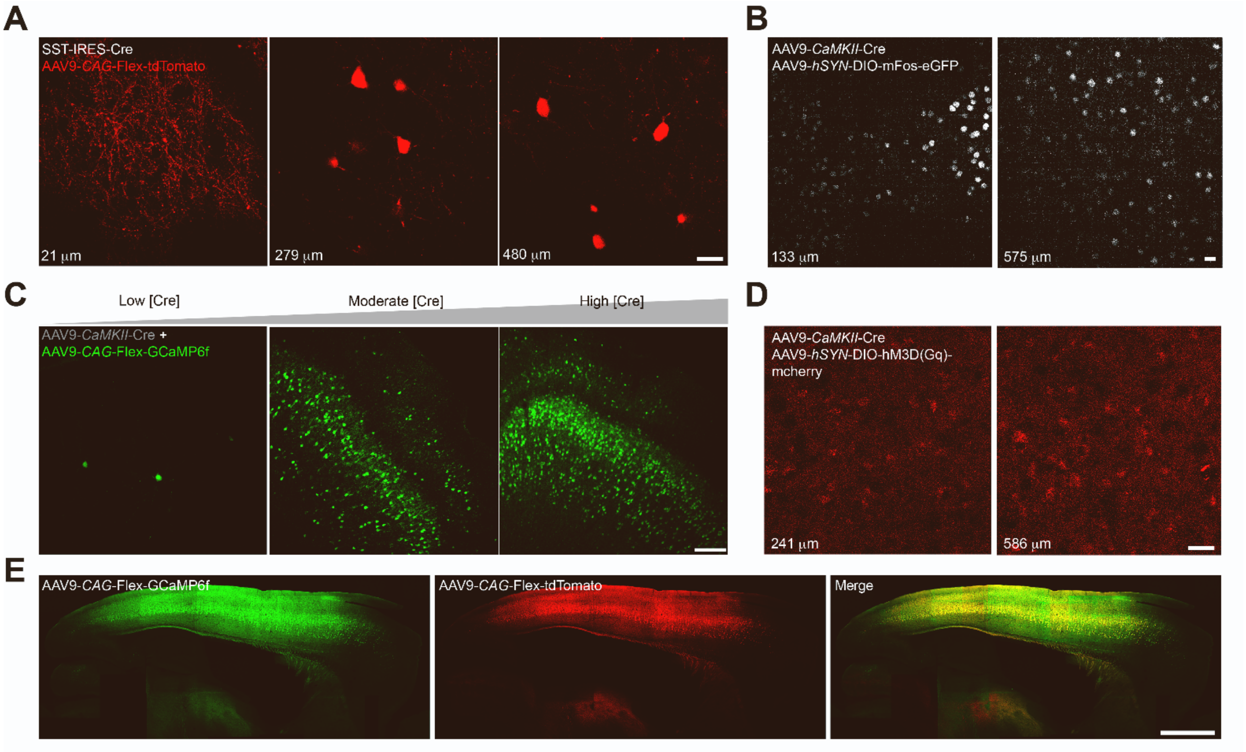

Figure S5. Neonatal AAV injection enables the expression of various genetically encoded sensors and reporters, related to Figures 3 and 4

(A) Two-photon images of neonatal SST-IRES-Cre mice injected with AAV9-*CAG*-Flex-tdTomato. Robust tdTomato expression was detected from superficial to deep cortical imaging planes. Scale bar, 20 μm.

(B) Two-photon images of neonatal mice co-injected with AAV9-*CaMKII*-Cre and AAV9-*hSYN*-DIO-mFos-eGFP reveal superficial to deep cortical labeling. Scale bar, 20 μm.

(C) Confocal images of coronal brain sections taken from mice co-expressing AAV9-*CaMKII*-Cre and AAV9-*CAG*-Flex-GCaMP6f in prefrontal cortex. AAV9-*CaMKII*-Cre concentration controls the density of Cre-dependent GCaMP6f labeling when coinjected with AAV9-*CAG*-Flex-GCaMP6f. Scale bar, 20 μm.

(D) Two-photon images of neonatal mice co-injected with AAV9-*CaMKII*-Cre and AAV9-*hSYN*-DIO-hM3D(Gq)-mCherry reveal superficial to deep cortical labeling. Scale bar, 20 μm.

(E) Confocal images of a sagittal brain section taken from a mouse co-expressing AAV9-*CaMKII*-Cre, AAV9-*CAG*-Flex-GCaMP6f, and AAV9-*CAG*-Flex-tdTomato targeted to primary somatosensory cortex. Scale bar, 1 mm.
